# Herzerkrankungen im Langzeitverlauf: Wie kann die psychosoziale Versorgung verbessert werden?

**DOI:** 10.1007/s00103-022-03516-z

**Published:** 2022-03-28

**Authors:** Karl-Heinz Ladwig, Julia Lurz, Karoline Lukaschek

**Affiliations:** 1 Klinik und Poliklinik für Psychosomatische Medizin und Psychotherapie, Klinikum rechts der Isar, Technische Universität München (TUM), Langerstr. 3, 81675 München, Deutschland; 2 Partnersite Munich, Deutsches Zentrum für Herz-Kreislauf-Forschung (DZHK), München, Deutschland; 3 Abteilung für Rhythmologie, Herzzentrum Leipzig, Universitätsklinik für Kardiologie, Leipzig, Deutschland; 4 Institut für Allgemeinmedizin, Klinikum der Universität München, LMU, München, Deutschland

**Keywords:** Herz-Kreislauf-Erkrankungen, Psychische Komorbiditäten, Versorgungsdefizite, Psychosoziale Stressfaktoren, Collaborative Care, Cardiovascular diseases, Psychological comorbidities, Care deficits, Psychosocial stress factors, Collaborative care

## Abstract

Herz-Kreislauf-Erkrankungen, zu denen in erster Linie die koronare Herzerkrankung (KHK), Herzrhythmusstörungen und die Herzinsuffizienz (HI) gehören, sind für die meisten Todesfälle und schwerwiegenden Krankheitsverläufe in der Europäischen Union verantwortlich. Das akute Geschehen steht meist im Vordergrund der klinischen Aufmerksamkeit. Dagegen existieren für den Langzeitverlauf dieser Krankheitsbilder kaum strukturierte Versorgungs- und Therapiekonzepte. Der vorliegende Beitrag gibt auf Grundlage einer Literaturrecherche eine Übersicht über die Langzeitfolgen und die Langzeitversorgung von Herzerkrankungen. Defizite in der psychosozialen Versorgung der Patienten und mögliche Lösungsansätze werden diskutiert.

Bei Patienten mit KHK ergeben sich aufgrund einer mangelhaften psychosozialen Langzeitversorgung häufig Probleme mit der Medikamententreue und der Einhaltung von Verhaltensempfehlungen. Psychische Komorbiditäten reduzieren die Lebensqualität und sind ein Antreiber für gesundheitsschädigendes Verhalten. Patienten mit Herzrhythmusstörungen geraten häufig in einen Teufelskreis aus wiederkehrenden körperlichen Beschwerden in Wechselwirkung mit Angst- und Panikattacken und der damit verbundenen Inanspruchnahme ambulanter, notärztlicher oder stationärer Versorgungseinrichtungen. Im Krankheitsverlauf einer Herzinsuffizienz wird eine klinisch bedeutsam wachsende Anzahl von Patienten mit Antidepressiva behandelt, deren Nutzen eher zweifelhaft ist.

Die erkennbaren Defizite der psychosozialen Langzeitversorgung von Herzerkrankungen können durch die verstärkte Anwendung systematischer kollaborativer Versorgungsmodelle von spezialisierten Versorgungseinrichtungen unter Einbeziehung von Hausärzten behoben und die Lebensqualität der Patienten verbessert werden.

## Abstract (en)

Cardiovascular diseases, which primarily include coronary artery disease (CAD), heart failure (HF) and cardiac arrhythmias, are the leading causes of death in the European Union and responsible for most of the serious courses of coronary disease. Acute events are usually the focus of clinical attention. In contrast, there are hardly any structured care and therapy concepts for the long-term course of these diseases. Based on a literature review, this article provides an overview of the long-term consequences and long-term care of heart diseases. Deficits in the psychosocial care of patients and possible solutions are discussed.

Patients with CAD often experience problems with medication adherence and compliance to behavioural recommendations due to inadequate long-term psychosocial care. Psychological comorbidities reduce the quality of life and are a driver for health-damaging behaviour. Patients with cardiac arrhythmias often get into a vicious circle of recurrent physical complaints interacting with anxiety and panic attacks and the associated use of outpatient, emergency, or inpatient care facilities. In the course of heart failure, a clinically significant growing number of patients are treated with antidepressants, the benefit of which is rather doubtful.

The apparent deficits in long-term psychosocial care of cardiovascular disease and the quality of life of patients could be improved through the increased use of systematic collaborative care models by specialised care facilities with the involvement of general practitioners.

## Einleitung

In Europa leben mehr als 100 Mio. Menschen mit Herz-Kreislauf-Erkrankungen, die mit hohen psychischen und physischen Belastungen einhergehen und von zentraler gesundheitsökonomischer Bedeutung sind [1]. Dies trifft in besonderem Maße auf die Krankheitsbilder koronare Herzkrankheit (KHK), Herzrhythmusstörungen (wie Vorhofflimmern und Kammertachykardien) und Herzinsuffizienz (HI) zu, die trotz unterschiedlicher Ätiologie eine Gemeinsamkeit haben: Das akute Geschehen steht im Vordergrund der klinischen Aufmerksamkeit und beansprucht Behandlungspriorität. Dies ist beispielsweise der Fall bei einer notfallmäßigen Krankenhausaufnahme aufgrund eines akuten Myokardinfarkts (AMI) oder einer dekompensierten Herzinsuffizienz sowie bei der Schockabgabe durch einen implantierbaren Kardioverter-Defibrillator (ICD) oder eine Vorhofflimmernepisode. Dagegen existieren für den Langzeitverlauf dieser Krankheitsbilder, die alle chronisch und damit im eigentlichen Sinne nicht heilbar sind, kaum strukturierte Versorgungs- und Therapiekonzepte.

Das Ziel der vorliegenden Arbeit ist es, auf Grundlage einer Literaturrecherche eine Übersicht über die Langzeitfolgen und die Langzeitversorgung von Herzerkrankungen zu geben und dabei Defizite in der psychosozialen Versorgung und mögliche Lösungsansätze aufzuzeigen.

## Langzeitverlauf und -versorgung von Herzerkrankungen

### Koronare Herzerkrankungen (KHK) und akuter Myokardinfarkt (AMI)

Bei einer KHK führt die progressive Lumeneinengung der Koronararterien zu einem Missverhältnis zwischen Sauerstoffbedarf und -angebot im Herzmuskel, das klinisch häufig mit Kurzatmigkeit und Brustschmerzen assoziiert ist und bei einer kritischen Lumeneinengung oder einem Komplettverschluss der Arterien in einen Herzinfarkt mündet. Lebensbedrohliche Folgeerkrankungen wie Herzschwäche oder Herzrhythmusstörungen können die Folgen sein. Allerdings lässt sich das Progressionsrisiko einer KHK durch die Bekämpfung verhaltensbedingter Risikofaktoren wie Rauchen, ungesunde Ernährung und Adipositas, Bewegungsmangel und schädlichen Alkoholkonsum verringern. Psychosoziale Faktoren, die akut (z. B. heftige Trauerreaktion, Ärgerausbruch), episodisch (z. B. Konflikte am Arbeitsplatz) oder chronisch (z. B. Persönlichkeitstyp D) auftreten können, tragen ebenfalls zum progressiven Verlauf einer KHK bei [2].

Auch wenn der Anteil der Todesfälle in Deutschland durch die KHK seit Jahren kontinuierlich sinkt, führt sie weiterhin (einschließl. des AMI) die Todesursachenstatistik an: Im Jahr 2017 betrug ihr Anteil an allen Todesfällen 8,3 % (*n* = 76.929), davon 53 % (*n* = 40.804) bei Männern [3]. Der sinkenden KHK-Mortalitätsrate steht die wachsende Zahl der Menschen gegenüber, die mit einer KHK leben: Mit einer Lebenszeitprävalenz von 9,3 % (95 % KI 8,4–10,3) bei 40- bis 79-Jährigen (*n* = 5901) – davon 4,7 % AMI-Patienten (95 % KI 4,0–5,5; [4, 5]) – gehört sie zu den wichtigsten Volkskrankheiten.

Im Langzeitverlauf eines AMI können sich für eine klinisch relevante Untergruppe psychische Komorbiditäten entwickeln. Hierzu zählen in erster Linie die Postinfarktdepression, aber auch Angstzustände und die posttraumatische Belastungsstörung (PTBS), die unabhängige Vorhersagefaktoren für ein nachfolgendes kardiales Morbiditäts- und Mortalitätsrisiko darstellen und die Lebensqualität der Betroffenen bedeutsam verringern [4–6].

Im Kontrast zur optimierten notfallmäßigen Behandlung der Patienten in der akuten Infarktsituation ist der Langzeitverlauf dieser Patienten wenig strukturiert, was mitverantwortlich ist für eine mangelhafte Adhärenz der Patienten bei der Einhaltung der Medikation, mehr aber noch für die Nichtbeachtung der Verringerung von Lebensstilrisiken mitverantwortlich [7]: In der jüngsten europaweiten EUROASPIRE-V-Erhebung^1^ erreichten 42 % der Patienten nicht den empfohlenen Blutdruckwert, 46 % der Patienten mit bekanntem Diabetes mellitus wiesen noch immer einen HbA1c-Wert von >7,0 % (53 mmol/mol) auf und der empfohlene Zielwert für Low-Density-Lipoprotein-(LDL-)Cholesterin von <1,8 mmol/L wurde nur bei 29 % der Patienten erreicht [8]. Angststörungen und Depressionen sind mit einer erhöhten Prävalenz von Bewegungsmangel, aktuellem und anhaltendem Rauchen und Fettleibigkeit assoziiert, verbunden mit einer geringeren Bereitschaft, Verhaltensänderungen umzusetzen [9].

Es ist daher wenig überraschend, dass AMI-Patienten im chronischen Verlauf ein erhöhtes Risiko für kardiovaskuläre Ereignisse (z. B. Reinfarkt, instabile Angina mit Indikation zur Revaskularisation, Schlaganfall oder Mortalität) aufweisen. In der prospektiven TIGRIS-Studie^2^ [10] waren dies rund 7 % der AMI-Patienten über einen zweijährigen Nachbeobachtungszeitraum und in einer bevölkerungsrepräsentativen schwedischen Registerstudie mit 100.879 eingeschlossenen Patienten erlitten ca. 21 % der Patienten während einer mittleren Nachbeobachtungszeit von fast 4 Jahren ein solches aversives Ereignis [11]. Diese Daten unterstreichen die Notwendigkeit zur Verbesserung der Versorgung von AMI-Patienten im Langzeitverlauf. Hierbei gilt es insbesondere zu beachten, dass neben den oben erwähnten affektiven Störungsbildern der sozioökonomische Status einen prägenden Einfluss auf den Krankheitsverlauf haben kann. Das Niveau des sozioökonomischen Status kann Ungleichheiten hinsichtlich Krankenhausressourcen und der persönlichen Einstellung von Patienten gegenüber ihrer Gesundheit widerspiegeln [12].

Kardiale Rehabilitationsprogramme (CRP) können die Sterblichkeit und das zukünftige Risiko eines erneuten Herzinfarktes reduzieren [13]. In Deutschland nehmen etwa 50 % der Patienten mit Herz-Kreislauf-Erkrankungen ihr Recht auf eine kardiologische Rehabilitation in Anspruch – immer noch mehr als im europäischen Durchschnitt, wo die Inanspruchnahme von CRP noch geringer ist, weswegen eine langfristige Betreuung der KHK-Patienten häufig auf Hausärzte zurückfällt [14, 15].

### Herzrhythmusstörungen

#### Vorhofflimmern (VHF)

VHF ist die häufigste anhaltende Herzrhythmusstörung und geht mit einer erhöhten Mortalität (1,9-fach bei Frauen und 1,5-fach bei Männern) und bedeutenden Morbidität (erhöhtes Schlaganfallrisiko bei bis zu 60 % der Betroffenen, kognitive Einbußen bzw. Demenz, erhebliche Einschränkung der Lebensqualität) einher [16, 17]. Das Lebenszeitrisiko für VHF steigt mit dem Alter bedeutsam an und beträgt ca. 37 % bei ≥55-jährigen Personen [17]. VHF ist in der Regel mit arterieller Hypertonie, Diabetes mellitus, KHK und/oder HI assoziiert. Katheterinterventionelle Verfahren bieten im Vergleich zu antiarrhythmischen Medikamenten zwar eine bessere Effektivität, jedoch sind Rezidive nach wie vor häufig [17].

Die Symptomatik ist extrem variabel: Bis zu 40 % der Betroffenen nehmen diese Rhythmusstörung (vermeintlich) nicht wahr [17]. Die Mehrheit jedoch leidet an einer Vielzahl von Beschwerden wie etwa an sehr unangenehm empfundenem arrhythmischen Herzrasen/Palpitationen, Schweißausbrüchen, Luftnot und Abgeschlagenheit. Nicht selten werden die körperlichen Symptome von Angst bis hin zu Panikattacken begleitet. Die ständige Angst vor dem Auftreten einer VHF-Episode kann zuweilen mehr Stress und Symptome verursachen als das VHF selbst [18]. Dies führt zu einem Teufelskreis aus wiederkehrenden körperlichen Beschwerden, häufig in Wechselwirkung mit den Angst- und Panikattacken und der damit verbundenen Inanspruchnahme ambulanter, notärztlicher oder stationärer Versorgungseinrichtungen. Negative Affektivität und psychosozialer Stress (bei bis zu 35 % der Patienten) tragen maßgeblich zur negativen Wahrnehmung des VHF sowie zur Verschlechterung der Lebensqualität bei [17, 18]. Hinzu kommen Ängste vor schwerwiegenden Nebenwirkungen insbesondere der blutverdünnenden und antiarrhythmischen Medikamente. Eine kompromittierte Einhaltung der vereinbarten Medikation (*Adherence to Treatment*) ist daher häufig die Folge. Vor dem Hintergrund der beschriebenen psychischen Komorbidität ist ein ausgeprägtes Vermeidungsverhalten (*Avoidance*) häufig. Die Betroffenen versuchen Trigger zu identifizieren, die ihr VHF auslösen, was jedoch meist ins Leere läuft und als frustrierend empfunden wird. Es gibt Hinweise, dass durch eine Verbesserung der depressiven Symptomatik auch eine Symptomlinderung des VHF gelingen kann [19]. Ein adäquates Risikofaktormanagement kann das Auftreten von VHF maßgeblich reduzieren; dieser Aspekt wird auch in der neuen VHF-Leitlinie berücksichtigt [17].

Für die Entwicklung des VHF spielt das autonome Nervensystem mit seinen komplexen Feedbackmechanismen eine herausragende Rolle [20], wobei eine sympathische-parasympathische Dysbalance entscheidend ist. Entspannungstechniken/Mentaltraining und Yoga können sich über eine Modulation autonomer Reflexe positiv auswirken [20, 21]. Hierzu gibt es allerdings bisher wenig verlässliche Daten. Ob der Einsatz von app-basierten Methoden effektiv in der Eindämmung des VHF ist, wird gegenwärtig in der *Mental-AF*-Studie^3^ geprüft.

Die psychische Notlage der VHF-Patienten wird von den betreuenden Ärzten häufig unterschätzt. Daher wäre eine systematische Erfassung der gesundheitsbezogenen Lebensqualität mittels *Patient-reported Outcome Measures (PROMs) *wünschenswert [17, 22]. Idealerweise sollten PROMs regelhaft bei Diagnosestellung, nach 6, dann nach jeweils 12 Monaten erhoben werden [17].

#### Kammertachykardien/-flimmern

In Europa sterben jährlich 350.000–700.000 Menschen an plötzlichem Herztod; als Ursachen liegen häufig eine KHK, eine HI oder angeborene Herzerkrankungen zugrunde. Entsprechend sind zum Teil junge, meist aber ältere Menschen betroffen. Der implantierbare Kardioverter-Defibrillator (ICD) ist in der Lage, solche Herzrhythmusstörungen zu detektieren und zu terminieren. Deutschland führt mit 350 Implantationen/1 Mio. Einwohner die europäische Implantationsstatistik an [23]. Die ICD-Implantation, ob sekundärprophylaktisch nach überlebtem plötzlichen Herztod oder primärprophylaktisch (bei hohem Risiko), stellt ein biografisch einschneidendes Lebensereignis dar. Hierbei geht es um 2 wesentliche Aspekte: Wie kommen die Patienten mit ihrer Krankheit zurecht *(Coping)* und inwiefern beeinflusst der ICD per se Körper und Psyche?

Ein entscheidender Faktor bei ICD-Patienten ist die hohe Prävalenz einer Multimorbidität (ca. 25 %), welche sowohl mit einer schlechteren Lebensqualität als auch mit einem erhöhten Mortalitätsrisiko assoziiert ist [24]. Während bei den jungen Patienten psychosoziale Faktoren wie Stress, Depressivität und finanzielle Sorgen im Vordergrund stehen, gewinnen mit zunehmendem Alter Gebrechlichkeit (*Frailty*), soziale Isolation und kognitive Einschränkung an Bedeutung [25]. Dabei ist bei ICD-Patienten die symptomatische HI der wichtigste klinische Prädiktor für eine eingeschränkte Lebensqualität und Angst [26].

Im Falle eines lebensbedrohlichen Rhythmusereignisses appliziert der ICD einen oder mehrere elektrische Schocks. Diese Schockabgaben werden häufig nicht als lebensrettend, sondern als traumatisierend erlebt [27] und können zu einer posttraumatischen Belastungsstörung (PTBS) führen, die mit einem 3,5-fach erhöhten Mortalitätsrisiko assoziiert ist, auch nach statistischer Kontrolle von Angst und Depression, welche wiederum häufig als Komorbidität auftreten [28]. Die Prävalenz von Angst und Depression bei ICD-Patienten liegt gemäß einer Metaanalyse durchschnittlich bei jeweils ca. 20 %, wiewohl größere, prospektive Studien mit Erfassung patientenorientierter Daten fehlen [29]. Angst ist nicht nur mit einem fast 2‑fach vermehrten Auftreten von Kammerarrhythmien, sondern auch mit einer 3‑fach erhöhten Sterblichkeit assoziiert [30]. Ein klinisch bedeutsamer Anteil der Patienten (ca. 37 %) leidet im Langzeitverlauf an herzbezogenen Ängsten (*Heart-focused Anxiety*; [31]). Nicht nur für diese Patienten ist der Gedanke, einen Anstieg der Herzfrequenz vermeiden zu müssen, allgegenwärtig und geht mit der Vermeidung von körperlicher Aktivität einher [32].

### Chronische Herzinsuffizienz

#### Stellenwert antidepressiver Behandlung im Langzeitverlauf bei HI-Patienten

Die chronische Herzinsuffizienz (HI) ist keine eigenständige Erkrankung, sondern beschreibt die eingeschränkte Leistungsfähigkeit des Herzens im Rahmen verschiedener Herzerkrankungen, die dazu führen, dass die Pumpleistung nicht mehr ausreicht, das vom Organismus benötigte Herzzeitvolumen bei normalem enddiastolischen Ventrikeldruck bereitzustellen. Unterteilt wird die HI nach der Auswurfleistung des Herzens (Ejektionsfraktion = EF), wobei zwischen HI mit reduzierter linksventrikulärer (LV) EF (HFrEF, LVEF < 40 %), HI mit mittelgradiger EF (HFmrEF, LVEF = 40–49 %) sowie HI mit erhaltener EF (HFpEF, LVEF > 50 %; [33]) unterschieden wird. In frühen Krankheitsstadien kompensieren gegenregulatorische Mechanismen den chronischen Verlauf der Erkrankung. Letztlich ist in der Mehrzahl der Fälle der progressiv-chronische Verlauf aber nicht aufzuhalten.

Häufige allgemeine Symptome einer HI sind verminderte Leistungsfähigkeit, Müdigkeit und Appetitlosigkeit. Je nachdem welcher Bereich des Herzens betroffen ist, treten außerdem Symptome wie Husten, Ödeme, Schlafstörungen und Atemnot auf. In späten Stadien der Erkrankung kann die Atemnot als Folge erhöhter Atemarbeit extrem traumatisierende Verläufe annehmen, bei denen die Betroffenen zwar atmen können, aber gleichzeitig das Gefühl haben zu ersticken. Daher ist es wenig überraschend, dass auch die psychische Belastung der HI-Patienten hoch ist und damit zur Kernsymptomatik der HI zu rechnen ist: Die Prävalenz von klinisch relevanten Depressionen bei HI-Patienten beträgt 21,5 % (Spannweite: 15,9–33,6 %; [34]) bis zu 42 % bei den schwerstkranken Patienten. Depression ist mit einem hohen Mortalitätsrisiko verbunden (Risk Ratio [RR] 2,1; 95 % KI 1,7–2,6) sowie mit signifikant mehr notfallmäßigen Krankenhausaufnahmen [34].

Die Bedeutung aversiver komorbider psychischer Belastungen gewinnt im klinischen Alltag an Aufmerksamkeit, was sich in einer Zunahme der Verordnung von Antidepressiva im Krankheitsverlauf zeigt [35]. Einen validen Einblick in die Lebensumstände von HI-Patienten mit einer antidepressiven Medikation in Europa [36] bieten die Daten eines dänischen Patientenregisters über den Zeitraum von 1997 bis 2010 mit 121.252 eingeschlossenen Patienten, die die Ersteinweisung mit der Indikation HI > 90 Tage überlebt hatten: Die Verordnung der psychopharmakologischen Behandlung der HI-Patienten nahm von 15,6 % (19.348) in der Erstuntersuchung über einen 5‑jährigen Nachverfolgungszeitraum auf 32 % bedeutsam zu. Nur bei 1 % der Patienten war eine Depression diagnostisch gesichert worden. Diese Daten belegen, dass Ärzte für die schwerwiegende psychische Komorbidität ihrer Patienten sensibilisiert sind. Die Symptomatik ist so ausgeprägt, dass im klinischen Alltag für die Verschreibung der Medikamente häufig auf eine formale Diagnosestellung verzichtet wird. Wie nützlich aber ist die antidepressive Medikation?

Die dänischen Registerdaten hierzu sind ernüchternd [36]: Sie zeigen, dass die Verschreibung von Antidepressiva mit einem signifikant erhöhten Gesamtmortalitätsrisiko (RR 1,34; KI 1,26–1,42) über einen 5‑jährigen Nachverfolgungszeitraum assoziiert ist. Eine aktuelle Metaanalyse bestätigt das erhöhte Gesamtmortalitätsrisiko (RR = 1,27; 95 % KI 1,21–1,34) einschließlich kardiovaskulären Todes (RR = 1,14; 95 % KI 1,08–1,20) bei HI-Patienten [37]. Ähnlich wie in der dänischen Registerstudie waren auch hier in einer Subgruppenanalyse die Medikamentenklassen Serotonin-Wiederaufnahmehemmer (SSRI; RR = 1,26; 95 % KI 1,19–1,32), trizyklische Antidepressiva (TZA; RR = 1,30; 95 % KI 1,16–1,46) und Serotonin-Noradrenalin-Wiederaufnahmehemmer (SNRI; RR = 1,17; 95 %; KI 1,08–1,26) alle mit einem erhöhten Gesamtmortalitätsrisiko assoziiert.

Die Alternative, den depressiven chronischen HI-Patienten Psychotherapie anzubieten, ist allerdings auch keine widerspruchsfreie Lösung: Forschung hierzu existiert kaum und wenn, dann im Wesentlichen für die kognitive Verhaltenstherapie (KVT), bei der sich nur mäßige Effektstärken für eine Depressionsbehandlung zeigen. Nach der Überzeugung von McPhillips et al. (2019) greifen überdies zentrale Paradigmen der KVT – wie z. B. die Auflösung negativer Vorstellungen durch Realitätstesten (*Cognitive Challenging*) – bei Patienten zu kurz, die einen so traumatisierenden progressiven Krankheitsverlauf erleiden, wie dies bei HI-Patienten fast regelhaft der Fall ist [38].

Was also tun? Unserer Auffassung nach bedarf es a) neuer Konzepte und Paradigmen in der therapeutischen Umsetzung, die zielgerichtet profitieren von dem Wissen über die neuropsychobiologischen stressinduzierten Funktionsabläufe, und b) neuer Horizonte in der psychosozialen Versorgung der schwerkranken HI-Patienten.

#### Neurohumorale Aktivierung bei der Herzinsuffizienz

In dem frühen, häufig noch symptomlosen Stadium der Erkrankung aktiviert der Körper kompensatorisch Adaptationsmechanismen, das erforderliche Herzzeitvolumen aufrechtzuerhalten, die mit dem Begriff „neurohumorale Aktivierung“ zusammengefasst werden. Bei chronischer Aktivierung tragen diese Mechanismen jedoch entscheidend zur Progression der Herzinsuffizienz bei. Hierzu zählt die Aktivierung des sympathischen Nervensystems (SNS), die anfangs zur Steigerung der Herzfrequenz und der Kontraktionskraft führt. Mit zunehmender Herzinsuffizienz steigt der Noradrenalinspiegel an. Gleichzeitig vermindert sich die Zahl der kardialen Betarezeptoren (*Downregulation*). Noradrenalin wirkt dadurch am Herzen immer weniger inotrop, erhöht aber den peripheren Widerstand (*Afterload*). Ein zweiter Mechanismus besteht in der Aktivierung des Renin-Angiotensin-Aldosteron-Systems (RAAS), das über eine erhöhte Produktion von Angiotensin II zur Vasokonstriktion und damit zur Erhöhung der Vorlast führt. Dieser Effekt wird durch das Mineralkortikoid Aldosteron verstärkt, das eine Natrium- und Wasserretention bewirkt und dessen Bioverfügbarkeit auch durch psychische Stressfaktoren beeinflusst wird [39].

Psychosoziale Stressfaktoren – allen voran Depression und Angst – sind eng mit einer neurohumoralen Aktivierung als zentrale Wirkmechanismen assoziiert und können als hinzukommende Faktoren (*Add-ons*) die Progression der HI weiter antreiben. Daten einer klinischen Fallkontrollstudie bei Patienten mit einer schwergradigen Depression (MDD) belegen, dass die MDD mit einer generellen Verschiebung der autonomen Bilanz zu einer sympathikotonen Prädominanz und einer gleichzeitigen Abnahme der parasympathischen Parameter [40] assoziiert ist. Überraschend verstärkten in dieser Studie SSRI oder (weniger ausgeprägt) SNRI dieses Ungleichgewicht weiter. Eine (depressionsinduzierte) autonome Dysregulation geht auch mit erhöhten Entzündungsparametern (z. B. Interleukin-6) einher [41], wie eine Untersuchung der Cardiovascular Health Study (*n* = 907; mittleres Alter 71,3 ± 4,6 Jahre; 60 % Frauen) belegt. Wenig überraschend erwies sich die Depression als ein robuster Prädiktor für ein kardiovaskuläres Mortalitätsrisiko (Hazard Ratio [HR] 1,88; 95 % KI 1,23–2,86).

Diese Befunde eröffnen eine neue Perspektive auf die Behandlung der komorbiden Depression bei der HI: Alles, was in der Lage ist, die autonome Bilanz zugunsten einer parasympathischen Regulation zu verschieben und dazu beiträgt, die subklinische Inflammation zu reduzieren, wird die Depression und damit auch die Progression der HI günstig beeinflussen. Umgekehrt wird eine nichtinvasive Vagusstimulation als ein neues vielversprechendes antidepressives Therapieprinzip diskutiert. So hat die ANTHEM-HF^4^-Studie zeigen können, dass eine andauernde hochintensive elektrische Vagusstimulation über einen mehrjährigen Zeitraum bei HI-Patienten in der Lage war, die autonome Funktion und die kardiale Stabilität zu verbessern und die Gefahr von ventrikulären Tachykardien zu reduzieren [42].

Patienten mit HI sind mit einem Krankheitsverlauf konfrontiert, der Episoden von Hoffnungslosigkeit und Verzweiflung einschließt. Die Aufmerksamkeit der behandelnden Ärzte für die psychische Komorbidität dieser Patienten nimmt zu – ist aber bei Weitem noch nicht ausreichend: In einer Untersuchung von insgesamt 3224 ambulanten HI-Patienten konnten die behandelnden Ärzte nur 14,1 % der zu dem damaligen Zeitpunkt depressiven Patienten identifizieren [43].

## Multiprofessionelle und sektorenübergreifende Ansätze für die Langzeitversorgung: Ein Blick in die Zukunft

*Collaborative Care*, ein Konzept der kooperativen Versorgung, beinhaltet im Kern die Idee, dass Interventionen zur Behandlung chronisch kranker Menschen multiprofessionell ausgestaltet sein sollten. Dies wird durch einen strukturierten, sektorenübergreifenden Versorgungsplan realisiert, mit dem die Kommunikation und Vernetzung zwischen den verschiedenen beteiligten Berufs- und Akteursgruppen unterstützt und gefördert wird. In der ambulanten Betreuung psychischer Komorbidität bei Herzpatienten liegt der Fokus auf der Koordination verschiedener, etablierter Therapieansätze (supportive Gesprächsangebote; ggf. Psychotherapie und/oder antidepressive Medikation) mit der kardiologischen Basistherapie ([4, 44]; Tab. 1).

**Table 1:** 

Modul	Inhalt	Zweck
*Systematisches Screening*	(1) Angst, Depression, PTBS bei Auftreten akuter kardialer Ereignisse sowie in regemäßigen Abständen (2–4×/Jahr) (2) PROM (gesundheitsbezogene Lebensqualität)	Frühzeitige Erkennung psychischer Komorbidität und Einleitung entsprechender therapeutischer Maßnahmen Optimierte Steuerung der therapeutischen Maßnahmen bzw. Behandlungspläne
*Patientenedukation*	Verständnis über Konzepte von Gesundheit und Krankheit, Bedeutung der Medikation und Maßnahmen	Stärkung der *Adhärenz*, Vermeidung unnötiger medizinischer Inanspruchnahme
*Risikofaktormanagement*	(1) Förderung körperlicher Aktivität/Herzsport, Ernährungsberatung, Nikotinkarenz (2) Einsatz von Entspannungsverfahren/Meditation	Reduktion von Morbidität und Mortalität, Verbesserung der Lebensqualität
*Psychosozialer Support*	(1) Supportive Gespräche (2) Selbsthilfegruppen (3) Psychotherapie (tiefenpsychologisch/systemisch), sofern indiziert (4) Ausgewählten Gruppen wie jungen ICD-Patienten sollte eine psychologische Betreuung zugänglich gemacht werden	Stärkung der *Adhärenz, *Reduktion psychosozialer Kompromittierung, Therapie psychischer Erkrankungen, Reduktion von Morbidität, Verbesserung der Lebensqualität
*Soziale Aspekte*	(1) Zügige Reintegration in die normalen Aktivitäten des täglichen Lebens, Eingliederung in einen ggf. veränderten beruflichen Alltag (2) Integration des sozialen Umfelds	Reduktion der Erwerbsunfähigkeit, Prävention negativer psychosozialer Einflüsse wie Einsamkeit, soziale Isolation

*ICD* implantierbarer Kardioverter-Defibrillator, *PTBS* posttraumatische Belastungsstörung, *PROM* Patient-reported Outcome Measures

Durch die Zunahme altersbedingter Multimorbidität und psychischer Komorbiditäten gewinnt die allgemeinmedizinische Primärversorgung immer mehr an Bedeutung. Ihre Aufgaben sind die langzeitige und umfassende Betreuung, z. B. bei chronisch (mehrfach) Kranken, und die Koordination verschiedener Disziplinen bzw. Professionen. Die Rolle der Fallmanager (Case Manager) – also derjenigen, die u. a. frühzeitig Symptome bei den Patienten dokumentieren, während der Behandlung monitoren, bei Bedarf eine kontinuierliche Unterstützung anbieten und den Arzt über Veränderungen informieren –, kann durch nichtärztliches Fachpersonal (z. B. med. Fachangestellte) abgedeckt werden [45]. Bosselmann et al. (2019) konnten zeigen, dass eine gemischte kollaborative Versorgung eine machbare, akzeptierte und wirksame Ergänzung in der Sekundärprävention der koronaren Herzkrankheit im deutschen Gesundheitssystem sein kann [46]. Durch eine gemeinsame Betreuung der Patienten durch Hausärzte und Spezialisten, durch hohe Leitlinientreue und durch multiprofessionelle sowie settingübergreifende Ansätze zur strukturierten Versorgung können Verlauf, Lebensqualität und/oder Krankenhausaufenthalte positiv beeinflusst werden [47].

Durch multimodale Ansätze aus Patientenedukation, Risikofaktormanagement mit körperlichem, aber auch mentalem Training und der damit verbunden Erlangung von Eigenverantwortung (*Self-empowerment*) kann eine gute Versorgung gelingen (z. B. [48]). Es scheint, dass psychoedukative Konzepte häufig mehr bewirken als formalisierte Psychotherapiekonzepte, eine psychopharmakologische Medikation oder medizinische Technologien. In der mit Frauen nach akutem kardialen Ereignis durchgeführten Studie SWITCHD^5^ ergab sich durch ein multimodal durchgeführtes Programm über 20 Sitzungen mit Fokus auf Stressmanagement, kognitiver Restrukturierung und Entspannungstechniken ein verbessertes Überleben von 7 % vs. 20 % nach 7 Jahren [49].

Herzpatienten und ihre Angehörigen dürfen nicht alleingelassen werden; sie müssen niederschwellig, aber dauerhaft betreut werden [50]. Hierzu bedarf es einer Stärkung und intensivierten Forschung von kollaborativen Versorgungsmodellen, aber auch im langfristigen chronischen Verlauf (insbesondere bei Herzinsuffizienzpatienten) einer Neuausrichtung der palliativen Versorgung, die deutlich früher und systematischer einsetzen muss, als es gegenwärtig der Fall ist.

## Footnotes

1 EUROASPIRE: European Survey of Cardiovascular Disease Prevention and Diabetes.

2 TIGRIS: „long-Term rIsk, clinical manaGement and healthcare Resource utilization of stable coronary artery dISease in post-myocardial infarction patients“.

3 Mental-AF-Studie: ClinicalTrials.gov Identifier NCT04067427; www.mental-af.de.

4 ANTHEM-HF: Autonomic Neural Regulation Therapy to Enhance Myocardial Function in Heart Failure.

5 SWITCHD: Stockholm Women’s Intervention Trial for Coronary Heart Disease.
